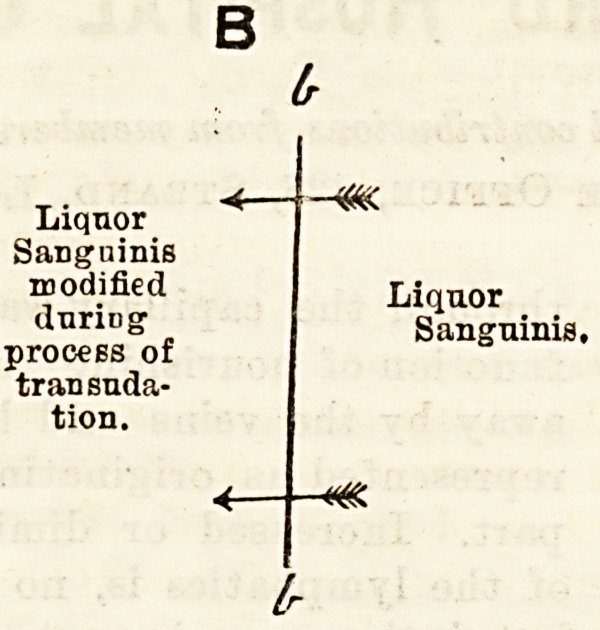# The Physiology of Œdema

**Published:** 1895-10-19

**Authors:** G. Munro Smith

**Affiliations:** Senior Assistant Surgeon Bristol Royal Infirmary, Professor of Physiology Bristol Medical School.


### Medical Progress and Hospital Clinics.

[*The Editor will be glad to receive offers of co-operation and contributions from members of the profession. All letters should be addressed to* The Editor, at the Office, 428, Strand, London, W.C.]

### THE PHYSIOLOGY OF ŒDEMA.

By G. Munro Smith, Senior Assistant Surgeon Bristol Royal Infirmary, Professor of Physiology Bristol Medical School.

An excess of fluid in the tissues outside the blood-vessels is found in many different conditions of the local and general circulations and of the excretory organs. It occurs when the blood-vessels are too full, when they are too empty, in arterial tension, in cases of weak heart and flabby arteries, in blocking or impediment which hinders the onward passage of venous blood, and in every variety of cardiac lesion. Yet in any one of these it may be absent. This alone would show that if it is brought about by mechanical causes these must be very complex. But the attempt to explain œdema this way has almost entirely failed.

Experiments on animals have thrown some light on the subject, or have, at least, given valuable negative evidence. But such experiments are very crude and imperfect representations of diseased conditions, and one cannot attach much importance to them. Ranvier, however, established one important fact, viz., that when the vena cava or the crural veins of a dog were ligatured and the return of blood hindered or stopped no œdema resulted. When, however, the nerves to the limb were cut and the arterioles dilated the tissues became rapidly dropsical. These two conditions, impeded flow in the veins and vaso-motor paralysis in the arteries, may exist together in certain diseased states, and if they do they are a sufficient explanation one would think. Unfortunately they appear to coexist occasionally without œdema supervening, and many considerations lead us to the conclusion that we must abandon any simple mechanical theory as untenable.

The physical conditions involved can be indicated by a simple diagram. A small arteriole, surrounded

Fig 1

by unstriped muscle and supplied by a vaso-motor nerve, is seen dividing into capillaries which run through the tissues *c.c.*, and are gathered into the small vein which only differs in structure from the capillary vessels by being slightly larger, and having, furthermore, some striped muscle and fibrous tissue. The fluid that normally bathes the tissues filters through the capillary walls, and, having fulfilled its function of nourishing the part, its excess is carried away by the veins and lymphatics, the latter being represented as originating amongst the cells of the part. Increased or diminished activity on the part of the lymphatics is, no doubt, an important factor, but it is more important in local than in general œdema, and there is no evidence to show that it is concerned in the dropsy of cardiac or renal origin.

In the above experiment of Ranvier's, when the veins are obstructed, why do not the tissues become water-logged? Three things may happen—(1) the supply to the part by the arterioles may be diminished and the pressure in the capillaries consequently lessened; (2) the lymphatics may take on double duty; or (3) the osmotic relations between the fluid inside and outside the capillaries may be altered.

The first of these, the contraction of the arterioles, probably plays an important part. Amongst other reasons for this being so these two may be given: (*a*) Dilatation of the blood-vessels by vaso-motor paralysis (as in the second part of Ranvier's experiment) causes œdema; and (*b*) contraction of the arterioles, whether due to the action of drugs or to abnormal conditions (for example, some form of Bright's disease) lessens the œdema. There is, apparently, a compensatory contraction of the arteriole at the point (*a*) when there is obstruction at (*b*). If the full complement of blood continued to flow it would be difficult to conceive how œdema could be prevented in venous obstruction, however active the lymphatics might be.

The third means of relief, viz., some alteration in the osmotic currents between the tissue fluids and the blood, will be considered directly.

It has been suggested that dropsy is really an inflammatory effusion, the result of an active inflammatory change in the connective tissues. This view, however, is negatived by the fact that inflammatory exudations contain a large quantity of albumen, whereas the fluid of œdema contains little. But it is to be noticed that the dropsy of cavities (peritoneum, &c.) is more like inflammatory exudations in this respect. If the physical condition of the two (dropsy of the tissues and dropsy of the closed serous cavities) be compared, an important difference is apparent, which may account perhaps for the smaller amount of albumen in the former. In the one case the liquor sanguinis filters through the capillary wall into a receptacle (*e.g.*, the pleura), where it accumulates without undergoing much change. In the other case the exuded liquid permeates amongst living cells, and mixes with the secretions of these and with the waste materials of their disintegration. If we represent the capillary wall by a line (*a.a.*), and let the space to the right of this line be the interior of the capillaries, and the space to the left be the tissues (in *A*), and the peritoneal or pleural cavity, &c. (in *B*), we have :—

In *B* the dropsical fluid will not differ much from the blood plasma; but in *A* it will be so modified by the tissues as to render a return osmotic current into the vessels highly probable, quite apart from any distinct "selective" influence.

In Bright's disease, moreover, where elimination is interfered with, and poisonous extractives are ever prone to accumulate, the endosmotic and exosmotic currents must be much altered.

Sir George Johnson has suggested that renal dropsy is an active secreting process exerted by the epithelium of the vessels themselves, and brought into action by the accumulation of noxious substances in the blood, and a great deal could be said in support of this view

Wooldridge found years ago (although the import ance of his researches, as Victor Horsley says, has not been sufficiently appreciated) that if tissue fibrinogen is injected into the circulation, and then a vein be obstructed, rapid œdema occurs, often accompanied by petechial hæmorrhages. Some direct toxic action is here at work, altering in some way the normal interchange of fluids. Again, water injected into the blood increases the tendency to œdema, and the same occurs if oxygen or albumen is diminished. This affords an explanation of the dropsy of cachexia.

Now, in the diseases in which dropsy is most common (cardiac and renal) some or all of these conditions are likely to exist. There is imperfect elimination and consequent accumulation of the products of metabolism; there is frequently a watery condition of the blood, disintegration of tissue, diminution of oxygen and waste of albumen.

How do these changes affect the capillary walls? The cells of which these are composed must be nourished like all other parts of the body, and this involves the powers of assimilation, of absorption, and of secretion. In other words, they are endowed with the property of selection. They can choose their appropriate food and reject the rest. They are capable (as Stricker has shown) of independent contraction.

We have to deal, then, not with a dead membrane, but with one endowed with all the manifold functions of life; and the processes of transfusion, filtration, and osmosis are modified by another factor, of unknown quantity, viz., vitality.

Professor Waymouth Reid, in some reports to the Scientific Grants Committee (see *British Medical Journal* for January 25th, 1890), has described some experiments on the skin of the frog, and its different behaviour to the passage of fluids when alive and dead. He found that osmosis is most rapid from without inwards in the living skin, whereas simple filtration (which is transfusion under pressure) takes place most easily from within outwards.

But when the skin has lost its vitality, osmotic transference is most rapid in the same direction as filtration, *i.e.*, from within outwards.

There are many other experiments (*e.g.*, Dutrochet on cœca of fowls, Claude Bernard on mucous membrane of stomach, &c.), which point to the same conclusion, and show the importance of vital action in modifying the various processes which go on in the animal body.

Applying the above experiments and reasoning to the blood-vessels, we may state without going beyond the tether of our knowledge that: (1) The mechanical theory cannot satisfactorily explain the phenomena of œdema. (2) The normal interchange of fluids between the liquor sanguinis in the capillaries and the tissues outside their walls is a vital process, and depends on the health of the cells composing these walls. (3) This interchange is deranged by diminution of oxygen, loss of albumen, excessive tissue change, imperfect elimination, watery conditions of the blood, and, in a word, abnormal metabolism. (4) These conditions are always present to some extent in general dropsy, and we may therefore look upon the one as (at all events partly) the cause of the other. (5) Œdema may therefore be attributed to (*a*) diminished vitality in the capillary wall, aided by (*b*) chemical changes in the blood and fluids altering the normal osmosic processes, and by (*c*) some mechanical conditions altering the pressure in the capillaries, generally venous obstruction combined with inactive or dilated arterioles.

## Figures and Tables

**Fig 1 f1:**
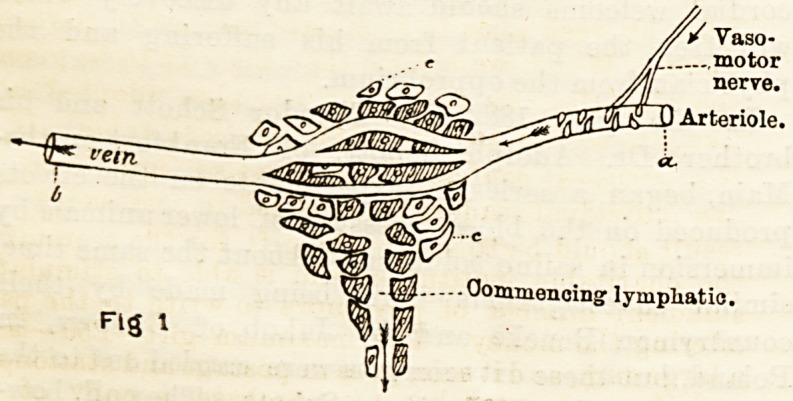


**Figure f2:**
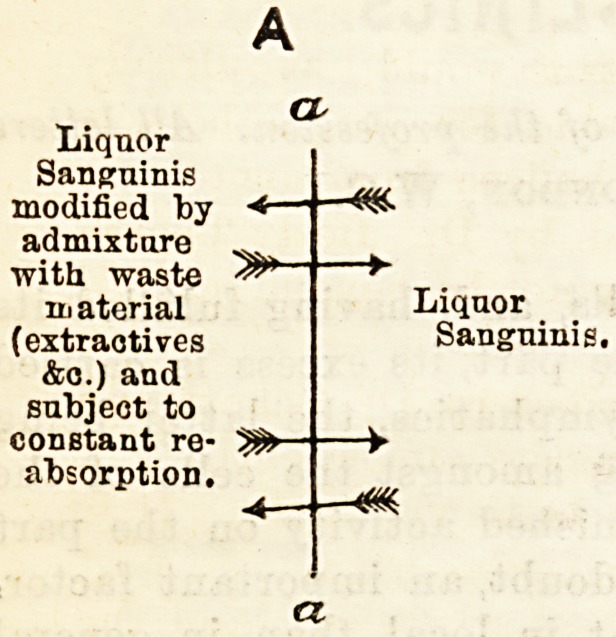


**Figure f3:**